# Maxillofacial haemorrhagic symptoms in emergency department patients: impact of antithrombotics

**DOI:** 10.1007/s00068-023-02428-0

**Published:** 2024-01-10

**Authors:** Pieter Date van der Zaag, Stephanie Geurts, Romke Rozema, Inge H. F. Reininga, Baucke van Minnen, REDUCTION study group members

**Affiliations:** 1 Department of Oral and Maxillofacial Surgery, University Medical Center Groningen, University of Groningen, Groningen, The Netherlands; 2 Faculty of Dentistry and Oral Medicine, University Medical Center Groningen, University of Groningen, Groningen, The Netherlands; 3 Department of Surgery, University Medical Center Groningen, University of Groningen, Groningen, The Netherlands; 4 Department of Trauma Surgery, University Medical Center Groningen, University of Groningen, Groningen, The Netherlands; 5 Emergency Care Network Northern Netherlands (AZNN), Northern Netherlands Trauma Registry, Groningen, The Netherlands

**Keywords:** Antithrombotics, Maxillofacial trauma, Maxillofacial haematoma, Epistaxis, Peri-orbital haematoma, Subconjuctival eccymosis, Intra-oral haematoma

## Abstract

**Purpose:**

To investigate the effect of antithrombotics on the occurrence of maxillofacial haemorrhagic symptoms, and to determine if these haemorrhagic symptoms are predictors of maxillofacial fractures.

**Method:**

A prospective cohort study was conducted of consecutive patients with maxillofacial trauma who had been admitted to the emergency department of four hospitals in the Netherlands. This study compared five haemorrhagic symptoms (peri-orbital haematoma, raccoon eyes, epistaxis, subconjunctival ecchymosis, and intra-oral haematoma) between patients not-using (NUA) and using (UA) of antithrombotics, and whether these maxillofacial haemorrhagic symptoms served as predictors for maxillofacial fractures.

**Results:**

Out of the 1005 patients, 812 (81%) belonged to the NUA group, and 193 (19%) to the UA group. UA patients exhibited higher frequencies of peri-orbital hematoma (54% vs. 39%, p < 0.001), raccoon eyes (10% vs. 5%, p = 0.01), and subconjunctival ecchymoses (16% vs. 7%, p < 0.001). In NUA, peri-orbital hematoma (OR = 2.5, p < 0.001), epistaxis (OR = 4.1, p < 0.001), subconjunctival ecchymosis (OR = 2.3, p = 0.02), and intra-oral hematoma (OR = 7.1, p < 0.001) were significant fracture predictors. Among UA, peri-orbital hematoma (OR = 2.2, p = 0.04), epistaxis (OR = 5.4, p < 0.001), subconjunctival ecchymosis (OR = 3.7, p = 0.008), and intra-oral hematoma (OR = 22.0, p < 0.001) were significant fracture predictors.

**Conclusion:**

Maxillofacial haemorrhagic symptoms were observed more frequently in the UA group than in the NUA group. However, in both groups, maxillofacial haemorrhagic symptoms appear to be predictors of maxillofacial fractures. Caution is warranted in attributing these symptoms solely to antithrombotic use during emergency department assessments.

**Supplementary Information:**

The online version contains supplementary material available at 10.1007/s00068-023-02428-0.

## Introduction

Antithrombotics inhibit haemostasis and are therefore prescribed to treat, and as a prophylactic measure against, thrombotic disorders such as cardiovascular diseases and cerebrovascular accidents [1]. In 2021, 1.9 million patients in the Netherlands, which is more than 10% of the Dutch population, were prescribed antithrombotics [2]. In addition, the World Health Organization predicts that the number of elderly people worldwide will continue to increase in the coming years. [3] This, in turn, will probably lead to a further increase in the number of antithrombotic users. This increase in antithrombotics use should be considered in trauma assessments and emergency department (ED) care.

It has been established that even minor trauma can cause haematomas in patients using antithrombotics [4]. Maxillofacial trauma is often associated with maxillofacial haemorrhagic symptoms such as peri-orbital haematoma and epistaxis [5, 6]. Maxillofacial haemorrhagic symptoms may indicate underlying severe injuries such as maxillofacial fractures [5, 7–9]. Nevertheless, it can be expected that there are differences in the occurrence of maxillofacial haemorrhagic symptoms after maxillofacial trauma between patients using antithrombotics and those not using antithrombotics. For example, the use of antithrombotics is known to increase the risk of epistaxis [10]. Previous research has also shown that patients using antithrombotics have a higher risk of orbital haemorrhage following orbital fractures [11]. Moreover, the use of antithrombotics at an advanced age is a predisposing factor for specific haematomas, such as retrobulbar haematomas following trauma [12]. However, studies have focused on specific haematomas, whereas the global relationship between antithrombotic use, maxillofacial trauma and maxillofacial haemorrhagic symptoms has not yet been established. Haemorrhagic symptoms may also influence the assessment of maxillofacial trauma in relation to maxillofacial fractures in the ED. Increased maxillofacial haemorrhagic symptoms may increase the suspicion of a maxillofacial fracture. On the other hand, maxillofacial fractures may be missed because haematomas are often attributed to antithrombotic use rather than the presence of a fracture. However, little research has been done on the probability of maxillofacial fractures in patients with maxillofacial haemorrhagic symptoms and antithrombotic use.

Thus, there appears to be a knowledge gap regarding the overall relationship between maxillofacial haemorrhagic symptoms and antithrombotic use in patients with maxillofacial trauma presenting at the ED. Therefore, the aim of this study was twofold; (1) to investigate the association between antithrombotic use and the incidence of maxillofacial haemorrhagic symptoms, and (2) to determine if these haemorrhagic symptoms, when assessed at the ED, are predictors of maxillofacial fractures in patients, both with and without antithrombotic use.

## Material and methods

### Study design

A prospective cohort study was performed. The cohort included all consecutive patients with midfacial or mandibular trauma who attended the ED of four hospitals in the North of the Netherlands between May 2018 and October 2019 [13]. The Institutional Review Board of the University Medical Centre Groningen (Groningen, the Netherlands) confirmed that the Medical Research Involving Human Subjects Act does not apply (METc code 2017/249) and local feasibility was approved by all the hospitals. The study was performed in compliance with the Declaration of Helsinki and the FEDERA (Foundation Federation of Dutch Medical Scientific Societies) code of conduct.

### Patient population

All consecutive patients aged 18 years and older presenting with midfacial and/or mandibular trauma at the ED were included. Patients who were using antithrombotics, but of who the type of antithrombotic was unknown, and patients who were using multiple antithrombotics simultaneously, were excluded.

The types of prescribed antithrombotics included Antiplatelet Agents (APAs), Vitamin K Antagonists (VKAs) and Direct Oral Anticoagulants (DOACs). The APAs included Acetylsalicylic acid, Ascal and Clopidogrel. The VKAs included Acenocoumarol and Phenprocoumon. The DOACs included Apixaban, Dabigatran, Edoxaban and Rivaroxaban.

### Data collection

The patients’ characteristics comprised sex, age, the use and type of antithrombotic, trauma mechanism, the type of maxillofacial trauma (i.e., midfacial and/or mandibular trauma), and the type of maxillofacial fracture (i.e., midfacial and/or mandibular fracture). The midfacial and/or mandibular injury was scored prospectively for each patient, followed by the presence or absence of associated maxillofacial haemorrhagic symptoms scored by the attending physician during the ED evaluation. Data regarding the presence and classification of midfacial and mandibular fractures were collected from the clinical information in the electronic medical records and radiographs.

### Outcome measures

The primary outcome was the incidence of maxillofacial haemorrhagic symptoms in maxillofacial trauma. The studied midfacial haemorrhagic symptoms consisted of peri-orbital haematoma, raccoon eyes, epistaxis and subconjunctival ecchymosis. Mandibular haemorrhagic symptoms included intra-oral haematoma. Precise definitions of the haemorrhagic symptoms are described in supplementary Table S1. Midfacial fractures were defined as any fracture of the frontal sinus, orbita, maxillary sinus, zygomaticomaxillary complex, nasoorbitoethmoid complex, nasal, Le Fort type fractures, and dentoalveolar fractures of the maxilla [14]. Mandibular fractures consisted of symphyseal and parasymphyseal fractures, corpus, angular, ramus, coronoid process, condyle and dentoalveolar fractures [15].

### Statistical analysis

The Statistical Package for the Social Sciences was employed for the data analysis (IBM Corp. Released 2015, IBM SPSS Statistics for Windows, Version 28.0). Categorical variables were presented as frequencies and percentages. Regarding the continuous variables, normally distributed variables were displayed as means and standard deviation (SD), and non-normally distributed variables as medians with interquartile range (IQR).

The first aim of this study was to evaluate the effect of antithrombotic use on the frequency of maxillofacial haemorrhagic symptoms. Therefore, the patient population was divided into a group of patients using antithrombotics (UA) and a group of patients not using antithrombotics (NUA). Pearson’s χ^2^ test or Fisher’s exact test were used to test the differences between the patient groups and the incidence of specific maxillofacial haemorrhagic symptoms. The Mann–Whitney U test was used to test differences in the non-normally distributed data, such as the total count of maxillofacial haemorrhagic symptoms per group. In addition, subgroup analyses were conducted to compare the incidence of maxillofacial haemorrhagic symptoms of the APA, VKA, and DOAC groups with that of the NUA group, using Pearson’s χ^2^ test or Fisher’s exact test.

The second aim of this study was to examine the likelihood of having a maxillofacial fracture when maxillofacial haemorrhagic symptoms were present. Patients clinically diagnosed as not having a fracture, and who therefore did not undergo radiography, were classified as having no fracture. Binary regression analysis (backward selection procedure) was performed to determine whether the presence of the maxillofacial haemorrhagic symptoms were predictors of a maxillofacial fracture for both groups. The threshold for the backward elimination of maxillofacial haemorrhagic symptoms was set at *p* = 0.157. Odds ratios (OR) were calculated with 95% confidence intervals (CI). Statistical significance was indicated with a p-value of < 0.05.

## Results

### Patient characteristics

The patients’ characteristics are presented in Table 1. A total of 1018 consecutive patients with maxillofacial trauma presented at the EDs between May 2018 and October 2019. Of these, four patients were excluded due to the use of an unknown antithrombotic type, and nine patients were excluded due to use of more than one antithrombotic agent. Among the remaining 1005 patients, 812 patients (81%) were in the NUA group, and 193 patients (19%) were in the UA group. The UA group was significantly older than the NUA group (median age of 77 vs. 55 years, respectively, *p* < 0.001). Midfacial trauma was reported significantly more often in the UA group than in the NUA group, respectively (97% vs. 90%, *p* = 0.002). Mandibular trauma (37% vs. 29%, *p* = 0.03) and mandibular fractures (23% vs. 7%, *p* = 0.009) were reported significantly more often in the NUA group than in the UA group, respectively.

**Table 1:** Patient characteristics

	Total	NUA	UA	p-value
Patients n (%)	1005 (100)	812 (81)	193 (19)	
Female gender n (%)	472 (47)	368 (45)	104 (54)	0.03*
Age in years (median (IQR))	56 (40)	50 (38)	77 (16)	< 0.001*
Trauma mechanism n (%)				
ADL at home	310 (31)	196 (24)	114 (59)	< 0.001*
Work	34 (3)	33 (4)	1 (< 1)	0.01*
Traffic	451 (45)	390 (48)	61 (32)	< 0.001*
Sport	34 (3)	31 (4)	3 (2)	0.12
Violence	123 (12)	118 (15)	5 (3)	< 0.001*
Fall	4 (< 1)	4 (< 1)	0 (0)	1.00^a^
Suicide attempt	3 (< 1)	3 (< 1)	0 (0)	1.00^a^
Other	36 (4)	30 (4)	6 (3)	0.69
Missing	10 (1)	7 (1)	3 (2)	0.42
Maxillofacial trauma n (%)^b^				
Midfacial trauma	916 (91)	729 (90)	187 (97)	0.002*
Midfacial fracture(s)	342 (37)	278 (38)	64 (34)	0.32
Mandibular trauma	356 (34)	301 (37)	55 (29)	0.03*
Mandibular fracture(s)	72 (20)	68 (23)	4 (7)	0.009*

UA, using Antithrombotics; NUA, not using antithrombotics; IQR, interquartile range; ADL, activities of daily living

*p < 0.05

^a^Fisher’s exact test performed

^b^Patients may have both midfacial and mandibular trauma

### Maxillofacial haemorrhagic symptoms

**Fig. 1:**
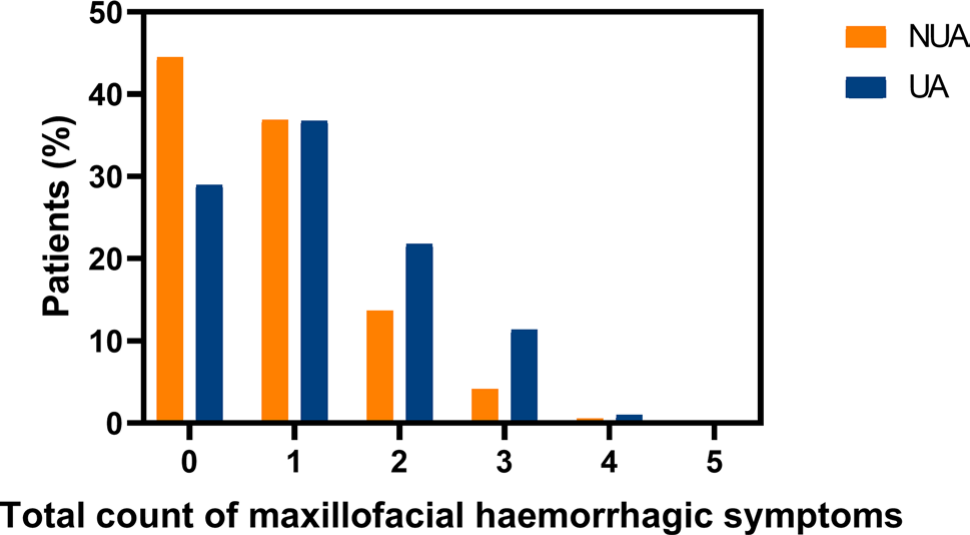
Total count of maxillofacial haemorrhagic symptoms per status of antithrombotic use. NUA, not using antithrombotics; UA, using antithrombotics

The incidence and total count of maxillofacial haemorrhagic symptoms are presented in Table 2 and Fig. 1. Peri-orbital haematoma was the most common (UA 54%, NUA 39%) and raccoon eyes was the least common (UA 10%, NUA 5%) in both the UA and NUA groups. In patients with midfacial trauma, peri-orbital haematomas (54% vs 39%, *p* < 0.001), raccoon eyes (10% vs. 5%, *p* = 0.01), and subconjunctival ecchymosis (16% vs. 7%, *p* < 0.001) were reported significantly more often in the UA group than in the NUA group, respectively. In patients with mandibular trauma, no significant difference was found in intra-oral haematoma occurrence (12% vs. 17%, *p* = 0.31) between the NUA and UA groups, respectively.

**Table 2:** Incidence of maxillofacial haemorrhagic symptoms

	NUA	UA	p-value
Peri-orbital haematoma n (%)	286 (39)	100 (54)	< 0.001*
Raccoon eyes n (%)	38 (5)	19 (10)	0.01*
Epistaxis n (%)	242 (34)	73 (40)	0.15
Subconjunctival ecchymosis n (%)	50 (7)	28 (16)	< 0.001*
Intra-oral haematoma n (%)	33 (12)	9 (17)	0.31

UA, using antithrombotics; NUA, not using antithrombotics

*p < 0.05

Regarding the total count, the UA group had significantly more haemorrhagic symptoms than the NUA group, both with a median of 1 (IQR_NUA_ = 1, IQR_UA_ = 1, *p* < 0.001).

### Maxillofacial haemorrhagic symptoms in the APA, VKA and DOAC subgroups

The incidence of maxillofacial haemorrhagic symptoms in the APA, VKA and DOAC subgroups is presented in Table 3. Among the midfacial trauma patients, peri-orbital haematomas (51% vs. 39%, *p* = 0.02) and subconjunctival ecchymosis (13% vs. 7%, *p* = 0.03) were reported significantly more often in the APA group than in the NUA group, respectively. Peri-orbital hematomas (67% vs. 39%, *p* < 0.001), raccoon eyes (13% vs. 5%, *p* = 0.03), epistaxis (54% vs. 33%, *p* = 0.003), and subconjunctival ecchymosis (23% vs. 7%, *p* = 0.001) were reported significantly more often in the VKA group than in the NUA group, respectively. No significant differences were found between the DOAC group and the NUA group. Among the patients with mandibular trauma, there were no significant differences in intra-oral haematoma occurrence between the APA, VKA and DOAC groups, and the NUA group.

**Table 3:** Incidence of maxillofacial haemorrhagic symptoms in the APA, VKA and DOAC groups compared with NUA-group

	NUA	APA	p-value	VKA	p-value	DOAC	p-value
Peri-orbital haematoma n (%)	286 (39)	56 (51)	0.02*	35 (67)	< 0.001*	9 (35)	0.64
Raccoon eyes n (%)	38 (5)	10 (9)	0.09	7 (14)	0.03*^a^	2 (8)	0.64^a^
Epistaxis n (%)	242 (34)	35 (32)	0.76	28 (54)	0.003*	10 (40)	0.52
Subconjunctival ecchymosis n (%)	50 (7)	14 (13)	0.03*	12 (24)	0.001*^a^	2 (8)	0.89^a^
Intra-oral haematoma n (%)	33 (12)	7 (24)	0.13^a^	0 (0)	0.40^a^	2 (22)	0.53^a^

NUA, not using antithrombotics; APA, antiplatelet agents; VKA, vitamin K antagonists; DOAC, direct acting anticoagulants

*p < 0.05

^a^Fisher’s exact test performed

### Maxillofacial fractures in patients with maxillofacial haemorrhagic symptoms

Table 4 shows which haemorrhagic symptoms were predictors of a midfacial or a mandibular fracture in both the NUA and UA patients.

**Table 4:** Predictors of a maxillofacial fracture per maxillofacial haemorrhagic symptom

Maxillofacial fracture per maxillofacial haemorrhagic symptom		NUA				UA			
		B	SE	OR (95% C.I.)	p-value	B	SE	OR (95% C.I.)	p-value
Midfacial fracture^†^	Peri-orbital haematoma	0.93	0.18	2.5 (1.8–3.6)	< 0.001*	0.78	0.38	2.2 (1.0–4.6)	0.04*
	Raccoon eyes					0.85	0.56	2.3 (0.8–7.1)	0.13
	Epistaxis	1.41	0.18	4.1 (2.9–5.9)	< 0.001*	1.69	0.37	5.4 (2.6–11.2)	< 0.001*
	Subconjunctival ecchymosis	0.84	0.37	2.3 (1.1–4.7)	0.02*	1.32	0.50	3.7 (1.4–9.9)	0.008*
Mandibular fracture^‡^	Intra-oral haematoma	1.96	0.39	7.1 (3.3–15.4)	< 0.001*	3.10	1.23	22.0 (2.0–247.1)	< 0.001*

UA, using antithrombotics; NUA, not using antithrombotics; B, logistic regression coefficients; SE, standard error; OR, odds ratio; C.I., confidence interval

*p < 0.05

^†^In the NUA group, 131 patients (18%) and in the UA group, 11 patients (6%) were clinically diagnosed as not having a midfacial fracture

^‡^In the NUA group, 56 patients (19%) and in the UA group, 12 patients (22%) were clinically diagnosed as not having mandibular fracture

Regarding the NUA group, peri-orbital haematoma (OR = 2.5 (95% CI 1.8–3.6), *p* < 0.001)), epistaxis (OR = 4.1 (95% CI 2.9–5.9), *p* < 0.001)) and subconjunctival ecchymosis (OR = 2.3 (95% CI 1.1–4.7), *p* = 0.02)) were significant predictors of midfacial fractures. Intra-oral haematoma (OR = 7.1 (95% CI 3.3–15.4), *p* < 0.001)) was a significant predictor of mandibular fractures in the NUA group.

Regarding the UA group, peri-orbital haematoma (OR = 2.2 (95% CI 1.0–4.6), *p* = 0.04)), epistaxis (OR = 5.4 (95% CI 2.6–11.2), *p* < 0.001)) and subconjunctival ecchymosis (OR = 3.7 (95% CI 1.4–9.9), *p* = 0.008)) were significant predictors of midfacial fractures, and intra-oral haematoma (OR = 22.0 (95% CI 2.0–247.1), *p* < 0.001)) was a significant predictor of mandibular fractures.

## Discussion

Due to the aging population, the number of patients using antithrombotics is increasing, as is the number of patients presenting at the ED with maxillofacial trauma [16, 17]. This study shows that the patients who used antithrombotics had more frequent maxillofacial haemorrhagic symptoms after maxillofacial trauma than patients who did not use antithrombotics. Several maxillofacial haemorrhagic symptoms were predictive of maxillofacial fractures in both patients using antithrombotics and not using antithrombotics.

Among the patients using antithrombotics (UA) and who had experienced midfacial trauma, the incidence of peri-orbital haematoma, raccoon eyes, and subconjunctival ecchymosis was significantly higher compared to the patients not using antithrombotics (NUA). There was also a trend towards a higher incidence of epistaxis in the UA group, though this difference was not statistically significant. In the mandibular trauma patient cohort, there was a higher, but not statistically significant, incidence of intra-oral haematomas in the UA group. Moreover, the UA group had, overall, significantly more haemorrhagic symptoms than the NUA group.

Previous research has indicated that hematomas occurring after traumatic brain injury may be influenced by the use of antithrombotic medications [18, 19]. However, this has not been established yet for maxillofacial trauma. One previous study stated that the use of antithrombotics is a predisposing factor for retrobulbar haematomas [12]. Another research reported that using antithrombotics results in a higher risk of orbital haemorrhage following an orbital fracture [11]. In this study, the incidence of epistaxis was only slightly different between the two groups (33%_NUA_ vs. 39%_UA_). This was not expected, given that epistaxis is a well-known side effect of antithrombotic use [20]. The similar incidence of epistaxis in both groups may be attributable to the vulnerable anatomic position of the nose in maxillofacial trauma, which makes epistaxis a common symptom in both groups [21, 22]. Possibly, the difference between the two groups should be looked into more in terms of recurrence or duration of the epistaxis [23].

When looking into the relation between the type of antithrombotic use (APAs, VKAs and DOACs) and maxillofacial haematomas, this study found that peri-orbital haematomas, raccoon eyes, epistaxis and subconjunctival ecchymosis were all significantly more common in patients using VKAs compared to patients not using antithrombotics. Only peri-orbital haematomas were reported significantly more often for the APA group than the NUA group, while no differences were found between the DOAC group and the NUA group. Multiple studies have found that DOACs demonstrate better outcomes than VKAs in cases with epistaxis, including hospital admissions, severity, recurrence, and treatment [24–27]. One study reported that VKA use is associated with an increased risk of spontaneous epistaxis, but that this was not observed for the APA patients [28]. This present study also showed a significantly higher incidence of epistaxis in the VKA patients and not in the APA patients. Only case reports have been found of subconjunctival ecchymosis and intra-oral haematoma associated with the use of antithrombotics [29, 30]. Several studies have shown that the use of VKAs results in more bleeding complications compared to the use of DOACs [31–34]. This is due to that the fact that DOACs directly inhibit thrombin (dabigatran) or factor Xa (rivaroxaban, apixaban, endoxaban) from exerting their anticoagulant effect, whereas VKAs inhibit a number of clotting factors [35]. This was also observed in this present study; patients using VKAs exhibited significantly more haemorrhagic symptoms in comparison to those not using antithrombotics, whereas this was not the case for patients using DOACs. An explanation for this is a lower compliance rate among DOACs users. A previous study showed that 51% of their patients using DOACs were non-compliant with the treatment due to its simplified management and the absence of international normalized ratio (INR) monitoring [34, 36]. This could potentially result in underreporting of the bleeding risks associated with DOACs. On the other hand, achieving INR stability is rarely achieved with standard VKA treatment, potentially leading to elevated INR levels [37]. This could raise the risk of major bleeding incidents, thereby leading to an overestimation of the bleeding risks associated with VKAs [37, 38]. The second research aim was to determine whether maxillofacial haemorrhagic symptoms are predictors of maxillofacial fractures in both UA and NUA maxillofacial trauma patients. In both studied groups, peri-orbital haematoma, epistaxis and subconjunctival ecchymosis were significant predictors of midfacial fractures. Additionally, raccoon eyes stood out as a predictor, although not significant, of midfacial fractures in the UA group but not in the NUA group. In both groups, intra-oral haematoma served as a significant predictor of mandibular fractures. Previous research has shown that raccoon eyes are a specific predictor of Le Fort fractures [9]. In this present study, raccoon eyes were not a significant predictor of midfacial fractures in both groups. This may be, because raccoon eyes are pathognomonic for Le Fort fractures and appear to have a limited predictive value for other types of midfacial fractures. Previous research reported intra-oral haematoma as a predictor of mandibular fractures [7]. As did this present research, strongly, for both patients using antithrombotics and not using antithrombotics. Thus, despite haemorrhagic symptoms being more frequent in the UA group, these symptoms remain viable predictors of maxillofacial fractures in both groups. Therefore, maxillofacial haemorrhagic symptoms should not be dismissed solely as the result of antithrombotic use when assessing patients at the ED.

### Strengths of this research

The main strength of this study is the large multicentre population, consisting of patients from the EDs of four different hospitals in the Netherlands. As a result, the study population and the assessing physicians cover a range of hospital trauma levels (levels 1–3), populations and geographical differences, which limits forms of selection bias. In addition, the effect of antithrombotic use on the presence of maxillofacial haematomas in maxillofacial trauma has been studied to a limited extent, and therefore this study adds to the knowledge base on the relationship between maxillofacial haemorrhagic symptoms and antithrombotic use.

### Limitations of this research

This study examined five types of maxillofacial haemorrhagic symptoms. There are undoubtedly other types and subtypes. Furthermore, there was no significant difference in the incidence of epistaxis and intra-oral haematoma between the two groups. This may indicate that the results are not fully generalizable to all types of maxillofacial haematomas and haemorrhagic symptoms. Moreover, this study used a dichotomous measure to reflect the presence or absence of haemorrhagic symptoms. Therefore, no distinction was made between the size and severity of the haematomas, and the persistence and recurrence of epistaxis was not assessed. In addition, low molecular weight heparins (LMWHs) were not included as a type of antithrombotic in this study, although this group has been investigated in other studies [39]. However, LMWHs are mostly used in a clinical setting, e.g. after surgery, which makes their presence less relevant in a study of trauma-related maxillofacial haemorrhagic symptoms [40]. In this study, the patients who were clinically diagnosed as having no fracture, and thus were not subjected to a maxillofacial radiograph, were categorized as having no fracture. This approach may have introduced bias, as midfacial and mandibular fractures might have been missed in these ED patients. However, clinical exclusion of fractures in non-severe maxillofacial injury is the standard practice for ED physicians. Therefore, the likelihood of missed maxillofacial fractures is low.

### Recommendations for further research

Further research is needed to prospectively evaluate all the maxillofacial haemorrhagic symptoms reported to the ED. Symptom size, such as haematoma size in square centimetres, and persistent and recurrent epistaxis, could also be recorded. This will enable a more comprehensive comparison between patients using and not using antithrombotics, not only in terms of the presence of haemorrhagic symptoms, but also in terms of their size and relevant characteristics. Moreover, the entire patient population with maxillofacial trauma should undergo radiographic imaging to eliminate the possibility of missing maxillofacial fractures.

## Conclusion

Patients using antithrombotics have moremaxillofacial haemorrhagic symptoms after maxillofacial trauma than patients not using antithrombotics. This difference is particularly evident in patients using VKAs. Although more frequent in patients using antithrombotics, maxillofacial haemorrhagic symptoms appear to be predictors of maxillofacial fractures in both groups. Therefore, when assessing trauma patients using antithrombotics in the emergency department, maxillofacial haemorrhagic symptoms should not be considered solely as the result of antithrombotic use.

### Supplementary Information

Below is the link to the electronic supplementary material.

Supplementary file1 (DOCX 21 kb)
