# Supplementary material for: Maxillofacial haemorrhagic symptoms in emergency department patients: impact of antithrombotics
Source: Eur J Trauma Emerg Surg. 2024 Jan 10;50(2):543–50. doi: 10.1007/s00068-023-02428-0 (PMC11035474; doi:10.1007/s00068-023-02428-0)
Supplement: Supplementary file 1 — Supplementary file1 (DOCX 21 kb) [file 68_2023_2428_MOESM1_ESM.docx]

Supplementary table S1 – Definition of the maxillofacial haemorrhagic symptoms ^14^

| **Midface** | |
| --- | --- |
| Peri-orbital haematoma | Any haematoma localized within or around the orbital or zygomaticomaxillary area that is not defined as raccoon eyes. A “*yes*” was scored if there was a peri-orbital haematoma. A “*no*” was scored if there was no peri-orbital haematoma. |
| Raccoon eyes | Bilateral ecchymosis or haematoma localized within and around the upper and/or lower eyelids. A “*yes*” was scored for cases with raccoon eyes. A “*no*” was scored if raccoon eyes were not present. |
| Epistaxis | A unilateral or bilateral active or past nosebleed. A “*yes*” was scored for cases with (past) epistaxis. A “*no*” was scored if there was no (past) epistaxis. |
| Subconjunctival haemorrhage | A sharply circumscribed bleeding or haemorrhage of the conjunctiva in one or both globes. A “*yes*” was scored if there was subconjunctival haemorrhage. A “*no*” was scored if there was no subconjunctival haemorrhage. |
| **Mandible** | |
| Intra-oral haematoma | Any intra-orally localized haematoma either in the mucosa or gingival tissue, including sublingual haematoma. A “*yes*” was scored if there was an intra-oral haematoma. A “*no*” was scored if there was no intra-oral haematoma. |
